# After Being Challenged by a Video Game Problem, Sleep Increases the Chance to Solve It

**DOI:** 10.1371/journal.pone.0084342

**Published:** 2014-01-08

**Authors:** Felipe Beijamini, Sofia Isabel Ribeiro Pereira, Felipe Augusto Cini, Fernando Mazzilli Louzada

**Affiliations:** Laboratório de Cronobiologia Humana, Departamento de Fisiologia, Universidade Federal do Paraná, Curitiba, Paraná, Brazil; Imperial College London, United Kingdom

## Abstract

In the past years many studies have demonstrated the role of sleep on memory consolidation. It is known that sleeping after learning a declarative or non-declarative task, is better than remaining awake. Furthermore, there are reports of a possible role for dreams in consolidation of declarative memories. Other studies have reported the effect of naps on memory consolidation. With similar protocols, another set of studies indicated that sleep has a role in creativity and problem-solving. Here we hypothesised that sleep can increase the likelihood of solving problems. After struggling to solve a video game problem, subjects who took a nap (n = 14) were almost twice as likely to solve it when compared to the wake control group (n = 15). It is interesting to note that, in the nap group 9 out 14 subjects engaged in slow-wave sleep (SWS) and all solved the problem. Surprisingly, we did not find a significant involvement of Rapid Eye Movement (REM) sleep in this task. Slow-wave sleep is believed to be crucial for the transfer of memory-related information to the neocortex and implement intentions. Sleep can benefit problem-solving through the generalisation of newly encoded information and abstraction of the gist. In conclusion, our results indicate that sleep, even a nap, can potentiate the solution of problems that involve logical reasoning. Thus, sleep's function seems to go beyond memory consolidation to include managing of everyday-life events.

## Introduction

In the past decades many studies have suggested that memory-consolidation is a function of sleep [1], [2]. These studies were performed with a whole night of sleep [3] (for review see [1]), in a split night with early and late nocturnal sleep [4], [5], or even with a single nap paradigm [6]. In animals, there is evidence of the replaying of memory traces during sleep, from neuronal [7] to molecular levels of plasticity [8]. Complementary, humans exposed to a virtual maze improve their performance on it after sleep [9]. Spatial orientation and navigation are known to be essentially hippocampus-dependent tasks [10] and hippocampal structures are also related to imagination and prediction [11]. Study results have found that sleep [12] and particularly slow-wave sleep [13], [14] plays a crucial role in the hippocampal-cortex dialog and consolidation of memories. Moreover, dreams during non-rapid eye movement (NREM) sleep may help to consolidate hippocampus-dependent memories. After playing a video-game, subjects whose dream mentation was related to the game, improved their performance on subsequent testing [15].

Sleep has also been shown to improve problem-solving and creativity. Studies have evaluated the effect of sleep on different subsets of problem solving such as generation of new associations [16], [17], insight [18], transition of implicit to explicit knowledge [19]–[21], abstraction of rules through grammar extraction, discrimination between rules [22] and creativity [23], [24]. Reactivations of hippocampal-dependent memories [25], [26] and non-hippocampal-dependent memories [27] indicate that sleep is important not only for stabilizing or strengthening memory traces, but also, to knowledge generalisation [28], [29]. Considering this evidence, it has been suggested that sleep's role is beyond the replay of memories during sleep.

The seminal work done by Wagner and colleagues in 2004 showed that sleep can increase the gain of insight. In that experiment, subjects learned a modified version of Number Reduction Task (NRT) with a hidden abstract rule. Discovering the hidden rule was considered insight. Subjects in the sleep group were twice as likely to discover the rule compared to the non-sleep group [18]. Beyond the gain of insight, the effect of sleep on the resolution of NRT was related to the transformation of implicit to explicit knowledge. Another study has suggested that SWS can be responsible for this transformation [19].

Additional support for the idea that sleep can boost problem-solving and creativity came from studies performed with the Remote Associates Test (RAT). Subjects were instructed to find a new word that was associated with three test words that did not seem to be related to each other. Rapid eye movement (REM) sleep improved the performance on the RAT [17].

In conclusion, several studies support the importance of sleep for memory consolidation, including information of future relevance [30] and also implementation of intentions [31]. However, to what extend does sleep play a role in problem-solving? Does sleep help to solve logical reasoning problems?

Here, we aimed to investigate the role of sleep in the ability to solve a mentally challenging problem.

## Materials and Methods

### Participants

Female students from the university campus were invited to take part in the study. Twenty-nine subjects (aged 21.55±3.57) finished all the steps of the study. All subjects were of age according to Brazilian laws. The study design was approved by the Research and Ethics Committee of the Biological Sciences Institute of the Federal University of Paraná, and our experiments were performed according to ethical standards of the Declaration of Helsinki. All participants gave written informed consent to voluntary participate in the study. Exclusion criteria were a history of neurological and psychiatric disorders, smoking and drug abuse, as well as the use of stimulant, depressor or hypnotic medication. Subjects were instructed to abstain from any food or beverage that contained caffeine 24 hours prior to the study.

### Experiment procedures

Following a screening process, subjects who met the inclusion criteria were invited to take part in the study. Besides answering a series of questionnaires (including the Epworth Sleepiness Scale (ESS), the Morningness-Eveningness Questionnaire (MEQ) [32], and an inquiry regarding sleep habits and experience with virtual games), participants were asked to wear an actigraph for a week, as well as to fill out sleep logs in order to monitor their sleep-wake cycle.

Upon arrival at the laboratory at noon, subjects were prepared for polysomnographic recordings and instructed how to play the game *Speedy Eggbert Mania®*.

The experiment consisted of a practice session (PS) before the incubation interval and a testing session (TS) after the incubation interval, whose existence the participants were not aware of. At the beginning of each session subjects were required to fill out the Karolinska Sleepiness Scale (KSS).

The practice session's duration varied according to each person's ability on the game. Subjects had up to 10 minutes to clear each level. When 10 minutes were complete and the subject still had not been able to clear that level, the practice session was terminated and a light meal was offered. Afterwards, during the 90-minute retention interval, subjects were assigned to either the sleep group (an afternoon nap) or the control group (quiet wakefulness).

In the testing session subjects were challenged by the same level that they had not been able to clear in the practice session and had up to 10 minutes to solve it.

**Figure 1:**
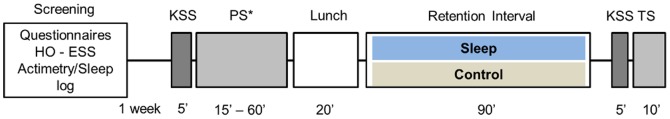
Study protocol. One week before the experimental day, subjects filled out questionnaires and the actigraphy begins. On the experimental day, practice session (PS) started at 1:00pm, after having a snack, subjects had an incubation interval of 90 minutes, starting around 1:30pm, which could be filled with sleep (Sleep group) or quiet wakefulness (Control group). The testing session (TS) was performed after the incubation interval, between 3:00pm to 4:00pm. At the beginning of each session subjects were required to fill out the Karolinska Sleepiness Scale (KSS).

The experimental procedures are schematically represented in Figure 1.

### Behavioural Task

The computer game *Speedy Eggbert Mania®* was used to probe problem-solving skills. Using only the mouse, players controlled the movement of an egg-shaped character named Blupi whose main goal is to reach a balloon that will take him to the next level. In order to reach the balloon, it is necessary to transport and/or move a collection of boxes available in a limited space within a 3D scenario. *Speedy Eggbert Mania®* has similarities with the well-known game *Sokoban*. In both games, logical reasoning is required, whereas procedural and declarative memory are kept to a minimum. All moves can be undone, providing the player with an opportunity to start over. As the difficulty increases, new objects show up in the scenario making navigation more complex.

For an objective analysis of each participant's performance, the computer screen was recorded during both the PS and TS. The number of levels played and the time spent on each level were computed.

**Figure 2:**
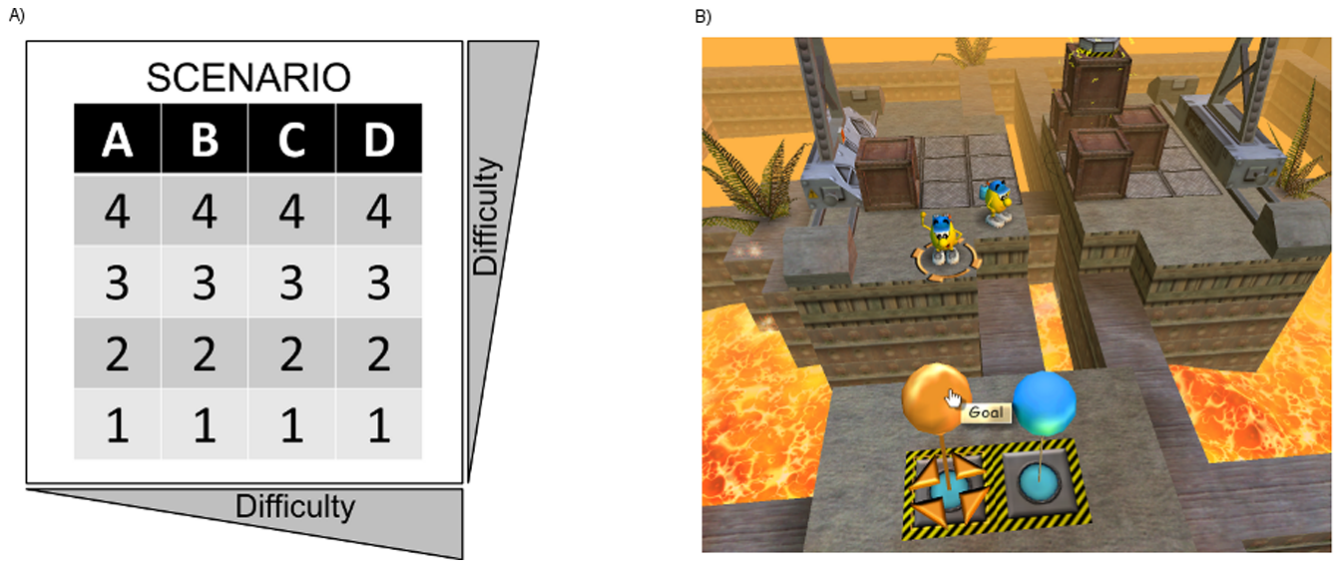
Speedy Eggbert Mania®. A) Representative schema of the levels of the game. The game is structured in four different scenarios (A to D) each one with four levels (1 to 4). Subjects started playing the first level (A1 to D1), then moved to the second level (A2 to D2) and so on. B) Example of one level (C3) of the game. Reprinted from Epsitec under a CC BY license, with permission from Epsitec, original copyright 2000.

Figure 2 presents a schema of the levels of the game (2A) and a sample of one level of the game (2B).

### Sleep recording and analysis

Actigraphy data were collected using wrist accelerometers. As mentioned above, subjects wore the device on their non-dominant wrist for one week. The following variables were considered for further analysis: sleep onset the night before experiment (SOB); wake-up time the day of the experiment (WUT); and total sleep time the night previous to the experiment (TST).

Polysomnography data were collected for both the sleep and control groups. To do so, electroencephalography (EEG) recordings (F3, F4, C3, C4, O1 and O2) were referenced to the mastoids, the electrooculography (EOG) electrodes were positioned in the canthus of the eyes and the electromyography EMG was recorded from chin electrodes. Data were stored in a computer and scoring was performed by a technician blind to the group and the subjects' performance. The scoring was done according to the 2007 Manual for Scoring from American Academy of Sleep Medicine (AASM) [33].

### Statistical analysis

To make sure both groups were in the same condition, *Student's t-tests* were performed with sleep-pattern variables obtained by actigraphy, Morningness-Eveningness Questionnaire MEQ score, ESS, and KSS scores. *Chi-square tests* were performed to compare the group and sleep stages effect on the solution of the problem. The p-value accepted as significant was ≤0.05.

## Results

**Figure 3:**
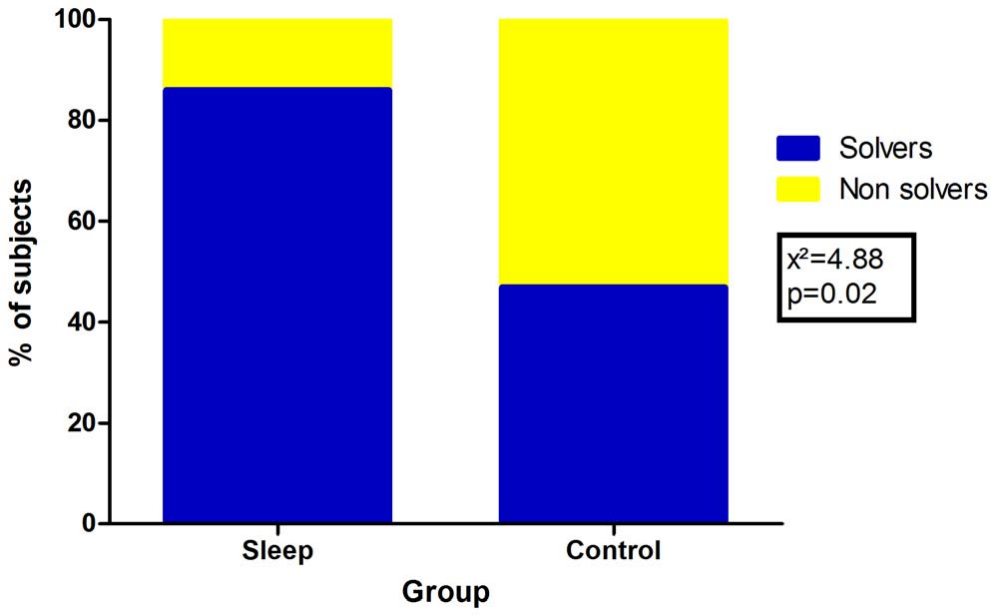
Effect of sleep on problem-solving. Bars show the percentage of subjects in each group. Blue represents the percentage of subjects who solved the problem; yellow represents the percentage of subjects who did not. Sleep group n = 14 (solvers n = 12, non-solvers n = 2). Control group n = 15 (solvers n = 7, non-solvers n = 8).

From the Control group, 7 out of 15 subjects solved the problem after the incubation interval. From the Sleep group, 12 out of 14 subjects solved the problem (X^2^ = 4.88 p = 0.02). This result is presented in Figure 3.

To evaluate if there were any confounding factors related to the performance, we analysed the demographic data and sleep patterns of each group (summarised in Table 1). Sleep and Control groups were balanced for age, body mass index and chronotype. In addition, there was no difference between groups in any sleep-pattern variable, and the sleepiness measured by the ESS was also similar between groups. The mean of score for MEQ chronotypes did not differ between groups.

**Table 1:** Demographic and sleep information from Control and Sleep Groups.

	Control	Sleep	p
**Subject**	15	14	
**Age**	20.73 (3.26)	22.43 (3.79)	0.20
**BMI**	23.36 (6.26)	23.22 (3.90)	0.94
**MEQ**	49.60 (11.49)	44.5 (14.86)	0.30
**ESS**	9.93 (3.89)	11.85 (4.09)	0.20
**KSS Before**	4.57 (1.22)	4.86 (1.26)	0.54
**KSS After**	5.07 (1.29)	4.26 (1.38)	0.17
**NLP**	6.66 (3.97)	4.50 (3.41)	0.12
**TST**	06:31 (01:19)	05:54 (01:25)	0.27
**SOB**	00:16 (01:26)	00:01 (01:13)	0.64
**WUT**	07:29 (01:26)	07:03 (01:51)	0.50

Means and standard deviations. Sleep patterns are expressed in hh:mm. BMI – Body mass index. MEQ – Morningness-Eveningness Score. ESS – Epworth sleepiness scale. KSS Before – Karolinska sleepiness scale before PS. KSS After – Karolinska sleepiness scale after incubation interval. NLP – mean of the Number of levels played. TST – Total sleep time the night before experiment. SOB - Sleep onset the night before experiment; WUT – Wake-up time the experimental day.

The architecture of the nap, obtained by polysomnography, is presented in Table 2.

**Table 2:** Nap architecture.

Sleep Duration		% Sleep Time		Sleep Latency:	
**Nap TST**	66.64 (16.99)	----	----	----	----
**WASO**	13.42 (15.25)	----	----	----	----
**Stage1**	15.32 (5.01)	**Stage 1 (%)**	26.36 (17.14)	**Stage1 (min)**	13.50 (7.02)
**Stage2**	37.17 (15.40)	**Stage 2 (%)**	54.69 (18.50)	**Stage 2 (min)**	19.36 (12.61)
**SWS**	9.71 (11.05)	**SWS (%)**	13.01 (20.68)	**SWS (min)**	46.50 (22.87)
**REM**	4.45 (4.89)	**REM (%)**	5.88 (6.33)	**REM (min)**	79.94 (10.11)

Means and Standard deviations. Sleep duration and Sleep Latency are expressed in minutes. Total Sleep Time during the Nap (Nap TST). WASO – Wake after sleep onset.

Table 3 presents the analysis of the relationship between sleep stages and solution. There was a significant effect of SWS sleep. Subjects who achieved slow-wave sleep were more likely to solve the problem. This effect was not observed for REM sleep. Furthermore, there is no statistical difference for dream recall frequency; subjects who remembered their dreams were not more likely to solve the problem than subject who did not remember their dreams. Only one subject's dream report was related to the task.

**Table 3:** Sleep stages and problem-solving.

	Solvers (n)	Non-Solvers (n)	X^2^	p
**SWS**	9	0	4.2	0.04
**No SWS**	3	2		
**REM sleep**	7	1	0.048	0.82
**No REM sleep**	5	1		
**Dream Recall**	6	0	1.75	0.18
**No Dream Recall**	6	2		

Subjects who achieved slow-wave sleep (SWS); Subjects who did not achieve slow-wave sleep (No SWS); Subjects who achieved rapid eye movement sleep (REM); Subjects who did not achieve rapid eye sleep (No REM); Subjects who remembered their dreams (Dream Recall); Subject who did not remember their dreams (No Dream Recall). Statistical comparisons are presented as the following, SWS *vs.* No SWS, REM *vs.* No REM, Dream Recall *vs.* No Dream Recall.

None of the subjects had ever played the *Speedy Eggbert Mania®* game. To test a possible confounding factor, the experience with other virtual games was compared. The distribution of subjects who self-reported being experts or naïve on virtual-games was similar between groups. The Sleep group had 8 experts and 6 naïve, while the Control group had 10 experts and 5 naïve (X^2^ = 0.27 p = 0.56). Also, we evaluated if the self-reported experience with virtual games would be able to predict the success in solving the problem. Twelve out of eighteen experts solved the problem, while seven out of eleven naïve did so (X^2^ = 0.02 p = 0.86); the self-reported game experience did not predict the success in solving the problem. Finally, we compared sleepiness at the testing session and the solution of the problem. We found no difference (t = 0.29 p = 0.64) between the sleepiness condition for Solvers (mean of KSS After 4.73±1.48) versus Non-Solvers (mean of KSS After 4.55±1.67).

## Discussion

Here we demonstrated that sleep, after being challenged by a logical reasoning problem, is better than quiet wakefulness to develop the solution. After sleeping, subjects were twice as likely to solve the problem when compared with subjects who spent the same amount of time awake. Our results add one more piece of evidence in favour of the idea that sleep can boost cognitive performance.

Previous studies had already shown that sleep boosts creativity [23], [24], problem solving [16], [17], and inspires insightful solutions in a numeric problem [18] and that these gains are related to specific sleep stages. There is evidence that REM sleep improves creativity. Cai and colleagues [17] showed that subjects who took a nap and engaged in REM sleep had better performance in the Remote Associates Test; also REM sleep is associated with consolidation of procedural memories [34]. Considering the possible association between REM sleep and problem-solving we evaluated if subjects who engaged in REM sleep stages were more likely to solve the problem. It is interesting to note that we did not find a direct relationship between REM sleep and improvement in problem-solving. When comparing the performance between subjects who did achieve REM sleep and the ones who did not, no significant difference was found. One reason for the lack of association between REM sleep and performance may be the characteristics of the problem presented here. It involves processing of spatial and visual information. Such information is known to be hippocampus-dependent [10], [11]. However, due to our experimental design on which 57% engaged in REM sleep, we cannot discard its role in problem-solving

There is evidence for the role of SWS in strengthening the consolidation of hippocampus-dependent memories [35]. In our experiment, the achievement of SWS was a predictor of the solution. Our results indicate that SWS sleep may be important for problem-solving in the conditions presented here. Furthermore, there are reports of SWS helping to implement an intention [31] and also the transfer of implicit to explicit knowledge [21]. Here, subjects were not informed that they would be tested on the same problem after sleep, but despite that, they still displayed an improvement in performance.

The napping protocol performed in our study has an important advantage; it is suppose to eliminate the circadian effects. However, this protocol may have some possible confounding factors, such as sleepiness in the control group. When subjective sleepiness was compared, no difference was found, for either practice or testing sessions. Also, sleep-patterns in the night previous to the experiment were similar in both groups. Complementary, we looked for associations between sleepiness and problem-solving, however, no relationship was found. This result is important to clarify that the sleep effect on problem-solving was not related to being more alert, but to the neural process underlying sleep [36].

A possible bias of the study is related to the differences in level difficulty at testing session. At practice session, subjects played freely until they were unable to solve one problem, which did not occur at the same level among subjects. In our experimental design every subject reached the most difficult level to them and was exposed to the same level again after the incubation interval. Consequently, the experimental design allowed a personalised approach, similarly to the study performed by Cai and colleagues [17].

One possible explanation for our results is that sleep can improve cognitive performance through an active process of memory consolidation and integration of recent experience into previous developed networks. Subjects who solved the problem may have a better understanding of the whole challenge and consequently were more capable of developing a strategy to solve the problem. Therefore, our results depict the beneficial role of a nap in complex-problem solving and provide further support to the hypothesis that sleep's ultimate goal is to improve an organism's fitness to its environment, by optimising the cognitive processes needed to successfully overcome everyday challenges.
